# Niobium Complexes Supported by Chalcogen-Bridged [OEO]-Type Bis(phenolate) Ligands (E = S, Se): Synthesis, Characterization, and Phenylacetylene Polymerization

**DOI:** 10.3390/molecules28062573

**Published:** 2023-03-12

**Authors:** Jing An, Akihiko Ishii, Norio Nakata

**Affiliations:** Department of Chemistry, Graduate School of Science and Engineering, Saitama University, 255 Shimo-Okubo, Sakura-Ku, Saitama 338-8570, Japan

**Keywords:** niobium, sulfur, selenium, tridentate ligand, X-ray diffraction analysis, polymerization, phenylacetylene

## Abstract

Trichloro niobium(V) complexes **3** and **4** with the sulfur- or selenium-bridged [OEO]-type bis(phenolate) ligands (E = S, Se) were synthesized and fully characterized on the basis of their NMR spectroscopic data and X-ray crystallographic analysis. In the crystalline state of **4**, the [OSeO]-core of the ligand was coordinated to the niobium center in a *fac*-fashion. The corresponding tribenzyl niobium(V) complexes **5** and **6** were also prepared by the reactions of **3** and **4** with 3 equivalents of PhCH_2_MgCl in toluene. The X-ray diffraction analysis of **6** revealed that the distorted six-coordinated niobium center incorporated in the [OSeO]-type ligand took a *mer*-fashion, and one benzyl ligand was coordinated to the niobium center by *η*^2^-fashion. Complexes **5** and **6** were tested for the phenylacetylene polymerization that produced poly(phenylacetylene)s (PPAs), oligomers, and triphenylbenzenes (TPBs) depending on the chalcogen atom in the [OEO]-type ligand.

## 1. Introduction

Phenoxide-based-chelating-[OXO]-type tridentate ligands (X = N, O, S) are widely employed as ancillary groups in the synthesis of early transition metal complexes because adequate substitution patterns allow for fundamental changes in the steric and electronic requirements of the metal center [1,2,3]. In particular, Group 4 metal complexes with [OEO]-type ligands have attracted attention, owing to their unique catalytic activity in the polymerization of olefins as well as the ring-opening polymerization of lactides and lactones [4,5,6,7,8,9,10,11,12]. Among such complexes, numerous studies on the syntheses and catalytic applications of Group 4 metal complexes bearing sulfur-bridged [OSO]-type bis(phenoxide) ligands have been reported up to now [13,14,15,16]. In a pioneering work, Kakugo and co-workers synthesized titanium complexes derived from [OSO]-type bis(phenol)s, and showed that, when activated with methylaluminoxane (MAO), these complexes could act as versatile pre-catalysts in the polymerization of ethylene and α-olefins, and the copolymerization of ethylene with styrene [17,18]. Janas et al. reported the preparation of [OSO]-type titanium complexes possessing a long sterically hindered auxiliary group such as 1,1,3,3-tetramethylbutyl group ortho to the phenoxide moiety [19,20,21,22]. These complexes, supported on MgCl_2_, showed extremely high activity for the ethylene polymerization (204,760 g mmol^−1^ h^−1^) upon activation with AlEt_2_Cl. Bercaw et al. presented the development of a new [OSO]-type bis(phenoxide) ligand which is bridged by a fused thiophene ring as a donor fragment [23]. The titanium and zirconium complexes with this ligand were found to promote propylene oligomerization or polymerization with good activity upon activation with MAO. Carpentier et al. also demonstrated the synthesis of a silyl-substituted bis(naphthoxy)-based [OSO]-type ligand incorporating a thiophene ring and the titanium complex derived thereof [24]. Recently, Mu et al. reported that titanium and zirconium complexes supported by CH_2_SCH_2_-brigded [OSO]-type bis(phenolate) ligands can work as suitable pre-catalysts with moderate-to-high performance in the polymerization of ethylene and copolymerization of ethylene with 1-hexene [25]. Furthermore, work on the [OSO]-type bis(phenolate) ligands was expanded to Group 5 metal complexes [26]. Despite such recent advances for the phenoxide-based-chelating-[OEO]-type ligands, there were only a few examples of the coordination chemistry utilized by a heavier selenium-bridged [OSeO]-type ligand. Furthermore, Group 5 metal complexes bearing the [OSeO]-type ligand have remained elusive to date.

As part of our ongoing research into developing sulfur-containing ancillary ligands, we reported several early transition metal complexes that contain an [OSSO]-type bis(phenolate) ligand based on a *trans*-1,2-cyclooctanediyl core [27,28,29,30,31,32,33,34]. In particular, the corresponding zirconium complexes activated by dried methylaluminoxane (dMAO) exhibited high catalytic performance in the polymerization of various α-olefins, resulting in the production of excellent isotactic poly(α-olefin)s [28,32]. We, therefore, wish to extend the use of the related chalcogen-based bis(phenolate) ligands to early transition metal complexes. Here, we present the preparations of several niobium(V) complexes incorporating sulfur- and selenium-bridged [OEO]-type ligands (E = S and Se) and an assessment of their catalytic behavior toward phenylacetylene polymerization.

## 2. Results and Discussion

**Scheme 1:**
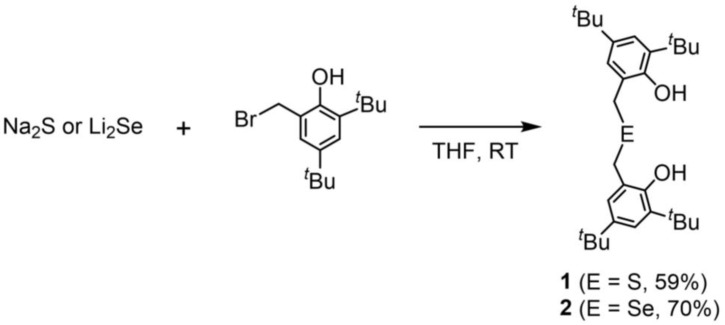
Synthesis of sulfur- and selenium-bridged [OEO]-type bis(phenol)s (**1**: E = S, **2**: E = Se).

Sulfur- and selenium-bridged [OEO]-type bis(phenol)s (**1**: E = S [25], **2**: E = Se) were readily prepared by the reactions of the 2-hydroxybenzyl bromide with Na_2_S or Li_2_Se in THF in 82% and 70% yields, respectively (Scheme 1). The structures of **1** and **2** were characterized by their spectroscopic data and elemental analyses.

**Scheme 2:**
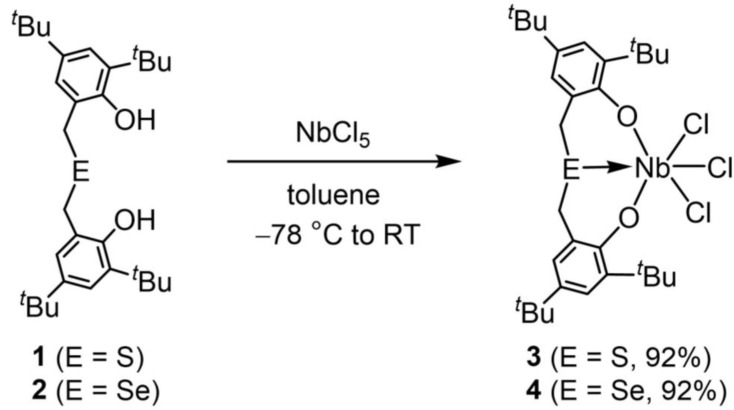
Synthesis of [OEO]-type trichloro niobium(V) complexes **3** and **4**.

The treatment of sulfur-bridged bis(phenol) **1** with NbCl_5_ in toluene afforded the corresponding trichloro niobium(V) complex **3** in 92% yield (Scheme 2). The ^1^H NMR spectrum of **3** exhibited the two sets of aromatic protons, *S*-benzyl protons as well as *^t^*Bu groups, indicating that complex **3** is formed as a mixture of *mer*- and *fac*-isomers. For example, two AB patterns for *S*-benzyl protons were observed at δ 3.02 and 3.52 (*J* = 14 Hz) and at δ 3.34 and 4.08 (*J* = 12 Hz) in a ratio of 0.9:1.0. To assign the chemical shifts for *mer*- and *fac*-isomers of **3**, the GIAO approximation at the B3PW91/6-311+G(2d, p) (LANL2DZ on Nb atom) level of theory was performed using the optimized structures of both isomers. As a result, two sets of two non-equivalent benzyl protons (δ 2.89, 3.68 (average 3.29), δ 4.48, 5.33 (average 4.91)) were predicted in *fac*-**3**, of which are slightly upfield-shifted compared to the two benzyl protons (δ 4.17, 4.93) calculated for *mer*-**3**. This indicates that the main product benzyl protons (δ 3.34 and 4.08) observed on the low-field side in the experimental data can be attributed to *mer*-**3**.

In a similar manner, selenium-bridged bis(phenol) **2** was treated with NbCl_5_ at −78 °C in toluene to lead to the formation of the corresponding trichloro niobium(V) complex **4** in 92% yield as a mixture of *mer*- and *fac*-isomers (Scheme 2). The structure of **4** was identified by NMR spectroscopic data based on the similarity to those of sulfur analog **3**. In the ^1^H NMR spectrum of **4**, two AB patterns due to the *Se*-benzyl protons appeared at δ 3.01 and 3.55 (*J* = 12 Hz) as a major fragment and at δ 3.55 and 4.23 (*J* = 11 Hz) as a minor in a ratio of 3.8:1.0. In the ^77^Se{^1^H} NMR of **4**, two signals were displayed at δ 149.0 (major) and 236.6 (minor). The major isomer showing the upfield-shifted benzyl-proton signals was tentatively assigned to *fac*-**4** by analogy with **3**.

**Figure 1:**
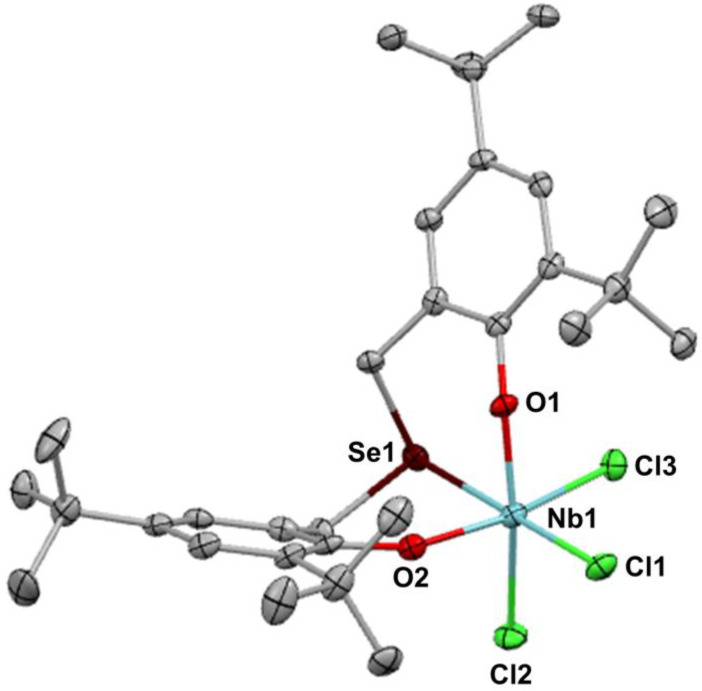
ORTEP of *fac*-**4** with thermal ellipsoids at 30% probability. All hydrogen atoms and one of the two crystallographically independent molecules of **4** in the unit cell are omitted for clarity.

The molecular structure of *fac*-**4** was finally determined by X-ray analysis, as shown in Figure 1. Complex **4** crystallizes in the triclinic space group *P*-1 with two crystallographically independent molecules per unit cell; the discussion will primarily focus on one molecule fragment of **4**. The central niobium atom in **4** possesses a distorted octahedral geometry in a facial fashion in which one selenium atom, one phenoxide oxygen atom, and two chlorine atoms are bonded to the metal center on the same plane. Selected bond lengths and angles of **4** are summarized in Table 1. The Nb1–Cl1 bond length in the *trans*-position of the selenium atom was 2.326(2) Å, which is slightly shorter than the other two Nb–Cl bond lengths [2.385(2) and 2.387(2) Å]. The Nb1–Se1 bond length was 2.7075(11) Å, which is considerably shorter than those found in the niobium(V) complexes supported by other selenide ligands, [2.7518(8)–2.8799(4) Å] [35,36,37,38]. The Nb–O bond lengths [1.888(5), 1.878(5) Å] fall in the range of those observed for related niobium(V) phenolato complexes (1.82–1.92 Å) [39,40]. Unfortunately, we could not isolate any crystals of the mer-**4**. The ^1^H NMR spectrum of the obtained crystals of *fac*-**4** resulted in the re-establishment of the same *mer*:*fac* ratio of 1.0:3.8, suggesting a readily equilibrium between *mer*- and *fac*-isomers in solution (see, Supplementary Materials).

**Scheme 3:**
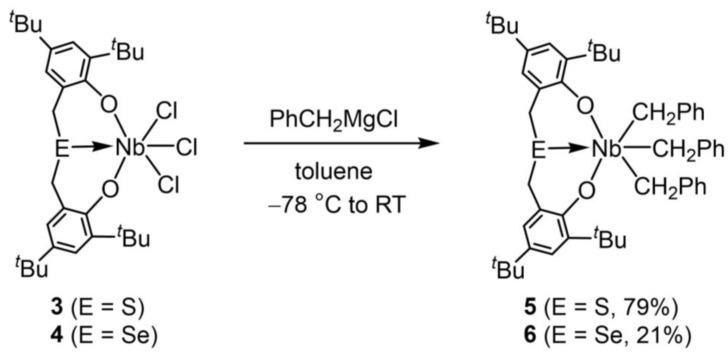
[OEO]-type tribenzyl niobium(V) complexes **5** and **6**.

Halogen-substituted niobium complexes are versatile precursors that can be functionalized by suitable nucleophilic substitution reactions to yield functionalized niobium complexes. Therefore, we next conducted the reactions of **3** and **4** with PhCH_2_MgCl as a nucleophile. Treatments of **3** and **4** with three equivalents of PhCH_2_MgCl proceeded at −78 °C in toluene to afford the corresponding tribenzyl niobium(V) complexes **5** and **6** in the form of orange crystals in 79 and 21% yields, respectively (Scheme 3).

In contrast to the starting trichloro complexes **3** and **4**, the NMR spectra of **5** and **6** showed the presence of only one structural isomer. In the ^1^H NMR spectra of **5** and **6**, the presence of two *^t^*Bu groups (δ 1.29 and 1.66 for **5**, δ 1.26 and 1.69 for **6**) and two sets of phenoxy groups (δ 6.93 and 7.53 for **5**, δ 6.95 and 7.54 for **6**) were observed. The AB pattern due to the *E*-benzyl protons appeared at δ 2.52 and 3.11 (*J* = 14 Hz) for **5** (E = S) and δ 2.55 and 3.27 (*J* = 14 Hz) for **6** (E = Se), whereas three benzyl protons directly bound to the niobium atom were displayed as magnetically inequivalent signals at δ 3.61, 3.80, and 4.16 for **5** and δ 3.64, 3.87, and 4.1 for **6**. In the ^77^Se{^1^H} NMR of **6**, a sharp signal resonated at δ 107.3.

**Figure 2:**
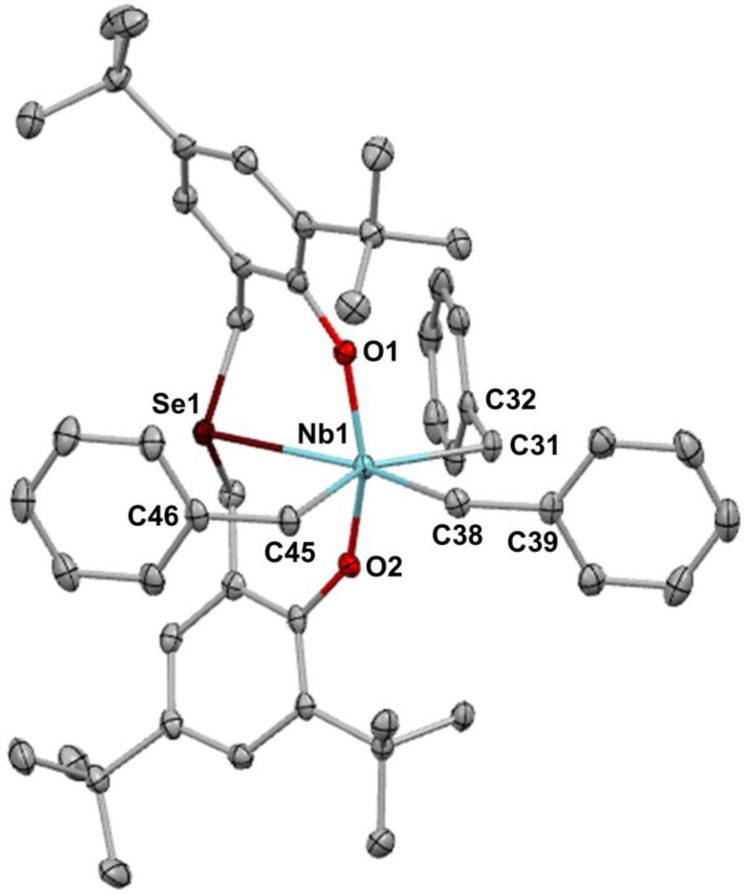
The ORTEP of *mer*-**6** with thermal ellipsoids at 50% probability. All hydrogen atoms and a molecule of hexane in the unit cell are omitted for clarity.

As illustrated in Figure 2, the crystal structure of **6** revealed that the selenium-bridged bis(phenolate) ligand wraps around the central niobium atom in a meridional fashion. Selected bond lengths and angles of **6** are summarized in Table 2. The niobium core has a distorted octahedral coordination sphere; the bond angles for Se1–Nb1–C38 [157.40(7)°], C31–Nb1–C45 [145.11(11)°], and O1–Nb1–O2 [154.05(8)°] deviate significantly from the ideal linear structure. In the three benzyl ligands coordinated to the niobium atom, the Nb1–C_benzyl_–C_ipso_ bond angle (Nb1–C31–C32) is 98.62(17)°, which is considerably smaller than the other two angles [Nb1–C38–C39: 128.4(2)°, Nb1–C45–C46: 114.2(2)°]. The distance between the ipso-carbon (C32) and niobium (Nb1) atoms is 2.844 Å, which is much shorter than the sum of the ionic radii of carbon and niobium atoms (3.16 Å), indicating that this benzyl ligand is coordinated to the niobium atom in an *η*^2^-fashion. The Nb1–Se1 bond length [2.8940(7) Å] in **6** is considerably elongated compared with that in **4** [2.7075(11) Å].

**Scheme 4:**
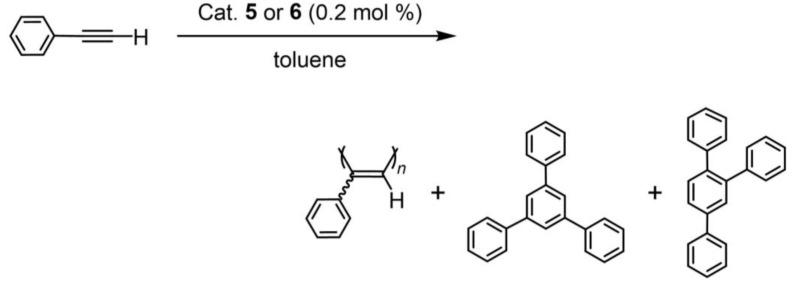
Polymerization of phenylacetylene catalyzed by **5** and **6**.

The polymerization of phenylacetylene (9.1 mmol) with **5** or **6** at the catalyst loading of 0.2 mol% in toluene was carried out under heating conditions (60 or 80 °C) (Scheme 4). The results are shown in Table 3. Under all reaction conditions, poly(phenylacetylene) (PPA) and oligomers were produced, together with 1,3,5- and 1,2,4-triphenylbenzenes (TPBs) as the cyclotrimerization products. The products were separated and purified using GPC, and the resulting PPA and oligomers were isolated as a red filmy material and an orange powder, respectively. TPBs were obtained as a colorless solid, and the formation ratios were established by the ^1^H NMR spectroscopy. When [OSO]-type complex **5** was used as a catalyst, the product yields were lower than those obtained with [OSeO]-type complex **6** (entries 1, 2, 4, 5). Raising the reaction temperature from 60 °C to 80 °C significantly increased the yields of PPA and TPBs (entries 2, 5). The yields of PPA and TPBs were also enhanced when the reaction time was extended from 3 to 24 h (entries 3, 6). The molecular weights (*M*_w_) and molecular weight distributions (PDI) of PPA and oligomers were analyzed by SEC (Table 3). The resulting PPA possesses a relatively narrow molecular weight distribution (PDI = 1.8–2.8), suggesting that the polymerization reaction proceeded at a single site. It also gave PPA with *M*_w_ of 9000–86,000 g mol^−1^. The molecular weight of PPA increased with the rising temperature from 60 °C to 80 °C during the polymerization with complex **5** (entries 1 and 2), whereas in the case of complex **6**, the molecular weight decreased by nearly half at the same conditions (entries 4 and 5). The stereochemistry of PPA was confirmed by ^1^H NMR spectroscopy. The resulting ^1^H NMR spectra of the obtained PPAs in CDCl_3_ exhibited a broad signal centered around δ 7.01, indicating the formation of a trans-cisoidal structure [41]. Neither the vinyl proton at δ 5.82 nor the aromatic protons at δ 6.65 and 6.91, which correspond to a cis-transoidal structure [41], were detected, suggesting the presence of PPAs with high trans content.

The analysis of the oligomeric components revealed the formation of oligomers ranging from hexamers to dodecamers with molecular weights ranging from approximately 500 to 1000 g mol^−1^. Additionally, the formation ratio of 1,3,5- and 1,2,4-TPBs was estimated through ^1^H NMR spectroscopy. The 1,3,5-TPB was furnished predominantly in the reaction at 60 °C, while 1,2,4-derivative emerged as a main product in reactions conducted at 80 °C.

## 3. Materials and Methods

General: All manipulations of air- and/or moisture-sensitive compounds were performed either using standard Schlenk-line techniques or in a GBJV080E300 Glovebox under an inert atmosphere of argon. Anhydrous solvents were purchased from Kanto Chemical Co., Inc. and used without further purification. Deuterated benzene (C_6_D_6_) and toluene (C_7_D_8_) were dried and degassed over a potassium mirror by the freeze–thaw cycle prior to use. Column chromatography was performed using a neutral Silica Gel 60 N (Kanto Chemical Co., Ltd., Tokyo, Japan). Melting points were determined on a Mel-Temp capillary tube apparatus and are uncorrected. ^1^H NMR was recorded on AVANCE-500 (500 MHz) or AV400+cryo (400 MHz) spectrometers, ^13^C NMR (100.6 MHz) was recorded on an AV400+cryo spectrometer, and ^77^Se (95.5 MHz) NMR spectra were obtained with an AVANCE-500 spectrometer. Elemental analysis was carried out at Molecular Analysis and Life Science Center, Saitama University. Preparative gel permeation liquid chromatography (GPC) was performed on an LC-918 (Japan Analytical Industry Co., Ltd., Tokyo, Japan) equipped with JAIGEL 1H and 2H columns (eluent: CHCl_3_). Molecular weights and molecular weight distributions of PPA and oligomers were determined against polystyrene standards by size exclusion chromatography (SEC) on an HLC-8220 GPC apparatus (Shimazu Corporation, Kyoto, Japan). THF was used as the eluent at a flow rate of 1.00 mL/min at 25 °C. The calibration was made by polystyrene standard EasiCal PS-1 (PL Ltd.).

Bis(2-hydroxy-3,5-di-*tert*-butylbenzyl) sulfide **1**: A THF solution (30 mL) of 3,5-di-*tert*-butyl-2-hydroxybenzyl bromide (5.00 g, 16.7 mmol) was added to a THF solution of sodium sulfide nonahydrate (2.00 g, 8.35 mmol), and the mixture was stirred for 16 h at room temperature. Then, the reaction mixture was extracted with dichloromethane, and the organic layer was separated, washed with water, and dried over anhydrous sodium sulfate. The solvent was removed under reduced pressure. The residue was reprecipitated with hexane to give **1** (2.10 g, 59%) as colorless crystals. Mp = 152–154 °C, ^1^H NMR (500 MHz, CDCl_3_) δ 1.29 (s, 18 H), 1.43 (s, 18 H), 3.75 (s, 4 H), 5.98 (s, 2 H), 6.95 (d, ^4^*J =* 2.0 Hz, 2 H), 7.27 (d, ^4^*J =* 2.0 Hz, 2 H). ^13^C{^1^H} NMR (101 MHz, CDCl_3_) δ 29.8, 31.6, 33.3, 34.2, 35.0, 121.7, 124.0, 125.2, 137.0, 142.5, 151.0. Anal. Calcd for C_30_H_46_O_2_S: C, 76.54; H, 9.85. Found: C, 76.58; H, 9.91.

Bis(2-hydroxy-3,5-di-*tert*-butylbenzyl) selenide **2**: Li_2_Se was prepared by a reduction of elemental selenium (0.83 g, 10.5 mmol) with lithium triethylborohydride (1.0 M, 100 mL, 26.0 mmol) in the THF solution at 0 °C. Then, a solution of 3,5-di-*tert*-butyl-2-hydroxybenzyl bromide (6.25 g, 20.8 mmol) in THF (20 mL) was added to the Li_2_Se-THF suspension at 0 °C, and the mixture was stirred for 19 h at room temperature. Then, the reaction mixture was extracted with diethyl ether, and the organic layer was separated, washed with water, and dried over anhydrous sodium sulfate. The solvent was removed under reduced pressure, and the residue was reprecipitated with hexane to give **2** (3.81 g, 70%) as colorless crystals. Mp = 144–146 °C, ^1^H NMR (500 MHz, CDCl_3_) δ 1.28 (s, 18 H), 1.42 (s, 18 H), 3.77 (s, ^2^*J*_Se-H_ *=* 5.0 Hz, 4 H), 5.54 (s, 2 H), 6.98 (d, ^4^*J =* 2.5 Hz, 2 H), 7.25 (d, ^4^*J =* 2.5 Hz, 2 H). ^13^C{^1^H} NMR (101 MHz, CDCl_3_) δ 25.1, 29.9, 31.6, 34.3, 35.0, 123.0, 123.9, 125.1, 136.9, 142.6, 151.0. ^77^Se{^1^H} NMR (95.4 MHz, CDCl_3_) δ 177.4. Anal. Calcd for C_30_H_46_O_2_Se: C, 69.61; H, 8.96; Found: C, 69.59; H, 8.92.

Reaction of 1 with NbCl_5_: A toluene solution (20 mL) of **1** (2.00 g, 4.26 mmol) was added to a toluene suspension (30 mL) of NbCl_5_ (1.15 g, 4.26 mmol) at −78 °C and the mixture was stirred for 15 h at room temperature. The solvent was removed under reduced pressure to give a crude dark powder. The obtained crude material was washed with hexane (10 mL) to give the corresponding trichloro niobium(V) complex **3** (2.61 g, 92%) as dark green crystals. Mp = 255–257 °C (decomp.). ^1^H NMR (500 MHz, C_6_D_6_) δ 1.22 (s, minor), 1.28 (s, major), 1.57 (s, minor), 1.77 (s, major), 3.04 (d, ^2^*J* = 13.5 Hz, minor), 3.36 (d, ^2^*J* = 12 Hz, major), 3.54 (d, ^2^*J* = 13.5 Hz, minor), 4.10 (d, ^2^*J* = 12 Hz, major), 6.74 (d, ^4^*J* = 2.5 Hz, minor), 6.99 (d, ^4^*J* = 2.5 Hz, major), 7.47 (d, ^4^*J* = 2.5 Hz, minor), 7.59 (d, ^4^*J* = 2.5 Hz, major). ^13^C{^1^H} NMR (101 MHz, δ, ppm, C_6_D_6_) δ 30.6, 31.0, 31.5, 31.6, 34.7, 34.8 35.3, 35.6, 36.1, 41.3, 123.8, 124.6, 124.9, 125.3, 126.21, 126.23, 127.9, 139.6, 147.1, 147.5, 156.7, 158.7. Anal. Calcd for C_30_H_44_Cl_3_NbO_2_S: C, 53.94; H, 6.64. Found: C, 53.85; H, 6.66.

Reaction of **2** with NbCl_5_: A toluene solution (10 mL) of **2** (500 mg, 0.97 mmol) was added to a toluene suspension (20 mL) of NbCl_5_ (313 mg, 1.15 mmol) at −78 °C, and the mixture was stirred for 17 h at room temperature. The solvent was removed under reduced pressure to give a crude dark powder. The obtained crude material was washed with hexane (10 mL) to give the corresponding trichloro niobium(V) complex **4** (633 g, 92%) as dark red crystals. Mp = 258–260 °C (decomp.). ^1^H NMR (500 MHz, C_6_D_6_) δ 1.22 (s, major), 1.29 (s, minor), 1.57 (s, major), 1.80 (s, minor), 3.11 (d, ^2^*J* = 12 Hz, major), 3.63 (d, ^2^*J* = 16 Hz, major), 3.65 (overlapped, minor), 4.27 (d, ^2^*J* = 11 Hz, minor), 6.82 (d, ^4^*J* = 2.5 Hz, major), 7.07 (d, ^4^*J* = 2.5 Hz, minor), 7.45 (d, ^4^*J* = 2.5 Hz, major), 7.59 (d, ^4^*J* = 2.5 Hz, minor) ^13^C{^1^H} NMR (101 MHz, C_6_D_6_) δ 30.5, 30.8, 31.0, 31.5, 34.7, 34.8 35.7, 36.2, 37.2, 41.3, 124.9, 125.0, 125.2, 125.6, 126.1, 126.3, 129.3, 140.1, 147.0, 147.3, 157.6, 159.9. ^77^Se{^1^H} NMR (95.4 MHz, C_6_D_6_) δ 149.0, 236.7. Anal. Calcd for C_30_H_44_Cl_3_NbO_2_Se: C, 50.40; H, 6.20. Found: C, 50.13; H, 6.12.

Reaction of **3** with PhCH_2_MgCl: A solution of PhCH_2_MgCl in ether (1.0 M, 0.5 mL, 0.50 mmol) was added to a toluene solution (10 mL) of **3** (106 mg, 0.159 mmol) at −78 °C and the mixture was stirred for 14 h at room temperature. Then, the resulting inorganic precipitates were filtrated off under argon atmosphere. The filtrate was evaporated to dryness and the residue was dissolved in hexane (5 mL) to remove insoluble material. The solvent was removed under reduced pressure, and the residue was recrystallized from the hexane solution. The resulting orange crystals were collected by filtration and washed with hexane to give the corresponding tribenzyl niobium(V) complex **5** (105 mg, 79%). Mp = 135–137 °C (decomp.). ^1^H NMR (500 MHz, C_6_D_6_) δ 1.30 (s, 18H), 1.66 (s, 18H), 2.54 (d, ^2^*J* = 13.5 Hz, 2H), 3.12 (d, ^2^*J* = 13.5 Hz, 2H), 3.61 (s, 2H), 3.80 (s, 2H), 4.15 (s, 2H), 6.56–6.75 (m, 8H), 6.94 (d, ^4^*J* = 2.5 Hz, 2H), 7.01–7.03 (m, 3H), 7.47 (t, ^3^*J* = 7.5 Hz, 2H), 7.53 (d, ^4^*J* = 2.5 Hz, 2H), 7.77 (d, ^3^*J* = 7.5 Hz, 2H). ^13^C{^1^H} NMR (101 MHz, C_6_D_6_) δ 31.0, 31.6, 34.6, 35.8, 40.2, 71.5, 78.1, 86.1, 122.8, 123.8, 123.9, 124.4, 125.6, 126.2, 128.64, 128.73, 128.84, 128.87, 128.9, 135.7, 138.0, 142.1, 145.0, 151.6, 153.8, 158.2.

Reaction of **4** with PhCH_2_MgCl: A solution of PhCH_2_MgCl in ether (1.0 M, 1.8 mL, 1.8 mmol) was added to a toluene solution (10 mL) of **4** (421 mg, 0.59 mmol) at −78 °C and the mixture was stirred for 14 h at room temperature. Then, the resulting inorganic precipitates were filtrated off under argon atmosphere. The filtrate was evaporated to dryness and the residue was dissolved in hexane (5 mL) to remove insoluble material. The solvent was removed under reduced pressure, and the residue was recrystallized from the hexane solution. The resulting orange crystals were collected by filtration and washed with hexane to give the corresponding tribenzyl niobium(V) complex **6** (111 mg, 21%). Mp = 152–154 °C (decomp.). ^1^H NMR (500 MHz, C_6_D_6_) δ 1.27 (s, 18H), 1.69 (s, 18H), 2.56 (d, ^2^*J* = 10.5 Hz, 2H), 3.29 (d, ^2^*J* = 10.5 Hz, 2H), 3.63 (s, 2H), 3.87 (s, 2H), 4.19 (s, 2H), 6.58–6.73 (m, 8H), 6.96 (d, ^4^*J* = 2.5 Hz, 2H), 6.99–7.08 (m, 3H), 7.46 (t, ^3^*J* = 7.5 Hz, 2H), 7.54 (d, ^4^*J* = 2.5 Hz, 2H), 7.79 (d, ^3^*J* = 9.0 Hz, 2H). ^13^C{^1^H} NMR (101 MHz, C_6_D_6_) δ 31.1, 31.6, 33.8, 34.6, 35.8, 72.6, 78.0, 85.7, 123.1, 123.5, 124.3, 124.7, 125.5, 126.1, 127.4, 128.8, 128.9, 129.02, 129.05, 135.4, 138.3, 142.8, 145.0, 153.1, 153.6, 159.1. ^77^Se{^1^H} NMR (95.4 MHz, C_6_D_6_) δ 107.3.

General procedure for phenylacetylene polymerization: To a suspension of **5** (16.7 mg, 0.02 mmol) or **6** (17.6 mg 0.02 mmol) in toluene (1 mL), phenylacetylene was added (1 mL, 0.93 g). The mixture was heated at 60 °C or 80 °C for 3 h or 24 h under an argon atmosphere. The reaction was quenched by methanol. The solvent was removed under reduced pressure, and the residue was subjected to silica-gel column chromatography (CH_2_Cl_2_) to remove insoluble organometallic materials. Then, the solvent was removed under reduced pressure, and the residue was purified by GPC.

X-ray diffraction analysis: Dark red single crystals of **4** were grown by slow evaporation of its saturated dichloromethane solution at 0 °C. Orange single crystals of **6** were grown by slow evaporation of their saturated hexane solution at 0 °C or room temperature. The data were collected on a Bruker SMART APEX II diffractometer at 100 K, using MoKα radiation (λ = 0.71073 Å). The structure was solved with SHELXT [42] using direct methods. The model was refined with SHELXL [43] using least-squares minimization. All hydrogen atoms were placed in geometrically idealized positions and constrained to ride on their parent atoms using the HFIX instruction.

Computational details: Geometries of *mer*- and *fac*-isomers of **3** were optimized with the gaussian-03 program [44] using the B3PW91/6-31+G(d) (LANL2DZ on Nb atom) level of theory. The GIAO approximation at the B3PW91/6-311+G(2d, p) (LANL2DZ on Nb atom) level of theory were performed using optimized structures of both isomers.

## 4. Conclusions

We have synthesized four niobium(V) complexes **3**–**6**, that incorporate sulfur- and selenium-bridged [OEO]-type ligands, where E = S and Se. The reaction of **1** or **2** with NbCl_5_ resulted in the corresponding trichloro niobium(V) complexes **3** or **4** as a mixture of *fac*- and *mer*-isomers. The treatment of **3** or **4** with three equivalents of PhCH_2_MgCl produced the tribenzyl niobium(V) complexes **5** or **6**, respectively, as a single structural *mer*-isomer. These complexes **5** and **6** were found to be effective catalysts in the phenylacetylene polymerization, providing poly(phenylacetylene)s (PPAs), oligomers, and triphenylbenzenes (TPBs). Further studies on the coordination chemistry of the [OEO]-type ligands are currently underway.

## Figures and Tables

**Table 1:** Selected bond lengths [Å] and bond angles [°] of **4**.

Bond Lengths	[Å]	Bond Angles	[°]
Nb1–O1	1.888(5)	Se1–Nb1–Cl1	177.56(6)
Nb1–O2	1.878(5)	O1–Nb1–Cl2	163.18(16)
Nb1–Cl1	2.326(2)	O2–Nb1–Cl3	161.35(17)
Nb1–Cl2	2.385(2)	Se1–Nb1–O1	81.68(15)
Nb1–Cl3	2.387(2)	Se1–Nb1–O2	82.16(16)
Nb1–Se1	2.7075(11)		

**Table 2:** Selected bond lengths [Å] and bond angles [°] of **6**.

Bond Lengths	[Å]	Bond Angles	[°]
Nb1–O1	1.901(2)	Se1–Nb1–C38	157.40(7)
Nb1–O2	1.915(2)	O1–Nb1–O2	154.05(8)
Nb1–C31	2.212(3)	C31–Nb1–C45	145.11(11)
Nb1–C38	2.346(3)	Se1–Nb1–O1	77.37(6)
Nb1–C45	2.275(3)	Se1–Nb1–O2	78.76(6)
Nb1–Se1	2.8940(7)	C32–C31–Nb1	98.62(17)
		C39–C38–Nb1	128.4(2)
Nb1...C32	2.844	C46–C45–Nb1	114.2(2)

**Table 3:** Polymerization of phenylacetylene catalyzed by **5** or **6** ^1^.

Entry	Cat.	Temp. [°C]	Yield of PPA [mg] ^3^	*M*_w_ [g mol^−1^] ^4^	PDI [*M*_w_/*M*_n_] ^4^	Yield of Oligomer [mg] ^3^	Yield of TPBs [mg] ^3^	Ratio of TPBs [1,3,5-/1,2,4-] ^5^
1	**5**	60	11	10,000	1.8	26	4	3.0/1.0
2	**5**	80	78	24,000	2.5	50	78	0.9/1.0
3 ^2^	**5**	60	80	25,000	2.5	31	51	1.0/1.0
4	**6**	60	141	86,000	2.4	31	30	1.1/1.0
5	**6**	80	218	44,000	2.8	42	90	0.5/1.0
6 ^2^	**6**	60	266	49,000	2.3	25	84	1.3/1.0

^1^ Conditions: cat. = 10 mM (0.2 mol%), phenylacetylene = 0.93 g (9.1 mmol), toluene = 1 mL, reaction time = 3 h. ^2^ Reaction time = 24 h. ^3^ Separated by GPC (CHCl_3_). ^4^ Determined by SEC using polystyrene standard for calibration. ^5^ Determined by ^1^H NMR.

## Data Availability

CCDC 2242067 (**4**) and 2242068 (**6**) contain the supplementary crystallographic data for this paper. These data can be obtained free of charge via www.ccdc.cam.ac.uk/data_request/cif (accessed on 15 February 2023), or by emailing data_request@ccdc.cam.ac.uk, or by contacting The Cambridge Crystallographic Data Centre, 12 Union Road, Cambridge CB2 1EZ, UK; fax: +44 1223 336033; E-mail: deposit@ccdc.cam.ac.uk.

## Supplementary Materials

The following supporting information can be downloaded at: https://www.mdpi.com/article/10.3390/molecules28062573/s1, All NMR spectra for complexes **3**–**6** and PPA, and crystallographic data for **4** and **6** in Crystallographic Information File (CIF) format. CCDC 2242067 and 2242068 also contain the supplementary crystallographic data for this paper.

## References

[1] Tshuva E.Y., Peri D. (2009). Modern Cytotoxic Titanium (IV) Complexes; Insights on the Enigmatic Involvement of Hydrolysis. Coord. Chem. Rev..

[2] Janas Z. (2010). Well-defined single-site thiobis(phenolate) Group 4 metal catalysts for heterogeneous olefin polymerization. Coord. Chem. Rev..

[3] (2015). Jean-François Carpentier. Rare-Earth Complexes Supported by Tripodal Tetradentate Bis(phenolate) Ligands: A Privileged Class of Catalysts for Ring-Opening Polymerization of Cyclic Esters. Organometallics.

[4] Tshuva E.Y., Versano M., Goldberg I., Kol M., Weitman H., Goldschmidt Z. (1999). Titanium complexes of chelating dianionic amine bis(phenolate) ligands: An extra donor makes a big difference. Inorg. Chem. Commun..

[5] Tshuva E.Y., Goldberg I., Kol M., Weitman H., Goldschmidt Z. (2000). Novel zirconium complexes of amine bis(phenolate) ligands. Remarkable reactivity in polymerization of hex-1-ene due to an extra donor arm. Chem. Commun..

[6] Tshuva E.Y., Goldberg I., Kol M., Goldschmidt Z. (2000). Living polymerization of 1-hexene due to an extra donor arm on a novel amine bis(phenolate) titanium catalyst. Inorg. Chem. Commun..

[7] Tshuva E.Y., Goldberg I., Kol M., Goldschmidt Z. (2001). Coordination Chemistry of Amine Bis(phenolate) Titanium Complexes:  Tuning Complex Type and Structure by Ligand Modification. Inorg. Chem..

[8] Chmura A.J., Davidson M.G., Jones M.D., Lunn M.D., Mahon M.F. (2006). Group 4 complexes of amine bis(phenolate)s and their application for the ring opening polymerisation of cyclic esters. Dalton Trans..

[9] Liang L.-C., Lin S.-T., Chien C.-C. (2013). Titanium Complexes of Tridentate Aminebiphenolate Ligands Containing Distinct N-Alkyls: Profound N-Substituent Effect on Ring-Opening Polymerization Catalysis. Inorg. Chem..

[10] Manne R., Miller M., Duthie A., Guedes da Silva M.F.C.G., Tshuva E.Y., Basu Baul T.S.B. (2019). Cytotoxic Homoleptic Ti (Iv) Compounds of ONO-Type Ligands: Synthesis, Structures and Anti-Cancer Activity. Dalton Trans..

[11] Shpilt Z., Manne R., Rohman M.A., Mitra S., Tiekink E.R.T., Basu Baul T.S., Tshuva E.Y. (2020). Homoleptic Ti[ONO]_2_ Type Complexes of Amino-acid-tethered Phenolato Schiff-base Ligands: Synthesis, Characterization, Time-resolved Fluorescence Spectroscopy, and Cytotoxicity against Ovarian and Colon Cancer Cells. Appl. Organomet. Chem..

[12] Nakayama Y., Watanabe K., Uetama N., Nakamura A., Harada A., Okuda J. (2000). Titanium Complexes Having Chelating Diaryloxo Ligands Bridged by Tellurium and Their Catalytic Behavior in the Polymerization of Ethylene. Organometallics.

[13] Fokken S., Spaniol T.P., Kang H.-C., Massa W., Okuda J. (1996). Titanium Complexes of Chelating Bis(phenolato) Ligands with Long Titanium−Sulfur Bonds. A Novel Type of Ancillary Ligand for Olefin Polymerization Catalysts. Organometallics.

[14] Sernetz F.G., Mülhaupt R., Fokken S., Okuda J. (1997). Copolymerization of Ethene with Styrene Using Methylaluminoxane-Activated Bis(phenolate) Complexes. Macromolecules.

[15] Natrajan L.S., Wilson C., Okuda J., Arnold P.L. (2004). Group 4 Complexes of Chelating Dianionic [OSO] Binaphtholate Ligands; Synthesis and Alkene Polymerisation Catalysis. Eur. J. Inorg. Chem..

[16] Tuskaev V.A., Gagieva S.C., Lyadov A.S., Kurmaev D.A., Zubkevich S.V., Shatokhin S.S., Simikin V.E., Mikhailik E.S., Golubev E.K., Nikiforova G.G. (2020). A Titanium(IV) Complex with an OSO-Type Ligand as a Catalyst for the Synthesis of Ultrahigh-Molecular-Weight Polyethylene. Pet. Chem..

[17] Miyatake T., Mizunuma K., Seki Y., Kakugo M. (1989). 2,2′-thiobis(6-tert-butyl-4-methylphenoxy)titanium or zirconium complex-methylalumoxane catalysts for polymerization of olefins. Makromol. Chem. Rapid Commun..

[18] Kakugo M., Miyatake T., Mizunuma K. (1990). Polymerization of styrene and copolymerization of styrene with olefin in the presence of soluble Ziegler-Natta catalysts. Studies in Surface Science and Catalysis.

[19] Janas Z., Jerzykiewicz L.B., Przybylak K., Sobota P., Szczegot K. (2004). Titanium Complexes Stabilized by a Sulfur-Bridged Chelating Bis(aryloxo) Ligand as Active Catalysts for Olefin Polymerization. Eur. J. Inorg. Chem..

[20] Janas Z., Jerzykiewicz L.B., Sobota P., Szczegot K., Wioeniewska D. (2005). Homo- and Heterometallic Aluminum and Titanium Complexes of Tridentate (OSO) Ligand: Synthesis, Structure, and Catalytic Activity. Organometallics.

[21] Janas Z., Jerzykiewicz L.B., Przybylak K., Sobota P., Szczegot K., Wioeniewska D. (2005). Titanium Complexes of Chelating, Dianionic O,S,O-Bisphenolato Ligands: Syntheses, Characterisation, and Catalytic Activity. Eur. J. Inorg. Chem..

[22] Wisniewska D., Janas Z., Sobota P., Jerzykiewicz L.B. (2006). Dimethyl Group IV Metal Complexes of the OSO-Type Ligand Bearing AlMe_2_ Moieties. Organometallics.

[23] Agapie T., Henling L.M., Dipasquale A.G., Rheingold A.L., Bercaw J.E. (2008). Zirconium and Titanium Complexes Supported by Tridentate LX2 Ligands Having Two Phenolates Linked to Furan, Thiophene, and Pyridine Donors: Precatalysts for Propylene Polymerization and Oligomerization. Organometallics.

[24] Kirillov E., Roisnel T., Razavi A., Carpentier J.F. (2009). Group 4 Post-metallocene Complexes Incorporating Tridentate Silyl-Substituted Bis(naphthoxy)pyridine and Bis(naphthoxy)thiophene Ligands: Probing Systems for “Oscillating” Olefin Polymerization Catalysis. Organometallics.

[25] Song T., He J., Liang L., Liu N., Li F., Tong X., Mu X., Mu Y. (2019). Titanium and zirconium complexes bearing new tridentate [OSO] bisphenolato-based ligands: Synthesis, characterization and catalytic properties for alkene polymerization. Dalton Trans..

[26] Redshaw C., Homden D.M., Rowan M.A., Elsegood M.R.J. (2005). Niobium-based ethylene polymerization procatalysts bearing di- and triphenolate ligands. Inorg. Chim. Acta..

[27] Nakata N., Toda T., Ishii A. (2011). Recent advances in the chemistry of Group 4 metal complexes incorporating [OSSO]-type bis(phenolato) ligands as post-metallocene catalysts. Polym. Chem..

[28] Ishii A., Toda T., Nakata N., Matsuo T. (2009). Zirconium Complex of an [OSSO]-Type Diphenolate Ligand Bearingtrans-1,2-Cyclooctanediylbis(thio) Core: Synthesis, Structure, and Isospecific 1-Hexene Polymerization. J. Am. Chem. Soc..

[29] Nakata N., Toda T., Matsuo T., Ishii A. (2012). Titanium Complexes Supported by an [OSSO]-Type Bis(phenolato) Ligand Based on a *trans*-Cyclooctanediyl Platform: Synthesis, Structures, and 1-Hexene Polymerization. Inorg. Chem..

[30] Nakata N., Toda T., Matsuo T., Ishii A. (2013). Controlled Isospecific Polymerization of α-Olefins by Hafnium Complex Incorporating with a trans-Cyclooctanediyl-Bridged [OSSO]-Type Bis(phenolate) Ligand. Macromolecules.

[31] Toda T., Nakata N., Matsuo T., Ishii A. (2013). Synthesis, Structure, and 1-Hexene Polymerization Catalytic Ability of Group 5 Metal Complexes Incorporating an [OSSO]-Type Ligand. ACS Catal..

[32] Saito Y., Nakata N., Ishii A. (2016). Highly Isospecific Polymerization of Silyl-protected ω-Alkenols with an [OSSO]-Type Bis(phenolato) Dichloro Zirconium(IV) Precatalyst. Macromol. Rapid Commun..

[33] Nakata N., Toda T., Saito Y., Watanabe T., Ishii A. (2016). Highly Active and Isospecific Styrene Polymerization Catalyzed by Zirconium Complexes Bearing Aryl-substituted [OSSO]-Type Bis(phenolate) Ligands. Polymers.

[34] Ishii A., Ikuma K., Nakata N., Nakamura K., Kuribayashi H., Takaoki K. (2017). Zirconium and Hafnium Complexes with Cycloheptane- or Cyclononane-Fused [OSSO]-Type Bis(phenolato) Ligands: Synthesis, Structure, and Highly Active 1-Hexene Polymerization and Ring-Size Effects of Fused Cycloalkanes on the Activity. Organometallics.

[35] Jura M., Levason W., Ratnani R., Reid G., Webster M. (2010). Six- and eight-coordinate thio- and seleno-ether complexes of NbF_5_ and some comparisons with NbCl_5_ and NbBr_5_ adducts. Dalton Trans..

[36] Galletti A.M.R., Pampaloni G. (2010). Niobium complexes as catalytic precursors for the polymerization of olefins. Coord. Chem. Rev..

[37] Chang Y.-P., Hector A.L., Light M.E., Reid G. (2016). Niobium tetrachloride complexes with thio-, seleno- and telluro-ether coordination—Synthesis and structures. Dalton Trans..

[38] Chang Y.-P., Hector A.L., Levason W., Reid G. (2017). Chalcogenoether complexes of Nb(V) thio- and seleno-halides as single source precursors for low pressure chemical vapour deposition of NbS_2_ and NbSe_2_ thin films. Dalton Trans..

[39] Matsuo T., Kawaguchi H. (2004). Tridentate Aryloxide Ligands: New Supporting Ligands in Coordination Chemistry of Early Transition Metals. Chem. Lett..

[40] Akagi F., Ishida Y., Matsuo T., Kawaguchi H. (2011). Synthesis and Reactivity of Niobium Complexes Having a Tripodal Triaryloxide Ligand in Bidentate, Tridentate, and Tetradentate Coordination Modes. Dalton Trans..

[41] Simionescu C.I., Percec V., Dumitrescu S. (1977). Polymerization of acetylenic derivatives XXX. Isomers of polyphenylacetylene. J. Polym. Sci. Polym. Chem. Ed..

[42] Sheldrick G.M. (2015). SHELXT—Integrated space-group and crystal-structure determination. Acta Crystallogr. Sect. A Found. Adv..

[43] Sheldrick G.M. (2015). Crystal structure refinement with SHELXL. Acta Crystallogr. Sect. C Struct. Chem..

[44] Frisch M.J., Trucks G.W., Schlegel H.B., Scuseria G.E., Robb M.A., Cheeseman J.R., Montgomery J.A., Vreven T., Kudin K.N., Burant J.C. (2003). Gaussian 03, Revision B. 04.

