# Supplementary material for: Niobium Complexes Supported by Chalcogen-Bridged [OEO]-Type Bis(phenolate) Ligands (E = S, Se): Synthesis, Characterization, and Phenylacetylene Polymerization
Source: Molecules. 2023 Mar 12;28(6):2573. doi: 10.3390/molecules28062573 (PMC10056720; doi:10.3390/molecules28062573)
Supplement: Supplementary file 1 [file molecules-28-02573-s001.zip › molecules-2282301-supplementary.pdf]

Supplementary Materials

Niobium Complexes Supported by Chalcogen-Bridged [OEO]-Type Bis(phenolate) Ligands (E = S, Se): Synthesis, Characterization, and Phenylacetylene Polymerization

Jin An, Akihiko Ishii, and Norio Nakata *

Department of Chemistry, Graduate School of Science and Engineering,

Saitama University, 255 Shimo-okubo, Sakura-ku, Saitama 338-8570, Japan

Table of Contents

Figure S1. ^{1}H NMR spectrum of compound **3**. S2

Figure S2. ^{13}C{^{1}H} NMR spectrum of compound **3**. S2

Figure S3. ^{1}H NMR spectrum of compound **4**. S3

Figure S4. ^{13}C{^{1}H} NMR spectrum of compound **4**. S3

Figure S5. ^{77}Se{^{1}H} NMR spectrum of compound **4**. S4

Figure S6. ^{1}H NMR spectrum of compound **5**. S4

Figure S7. ^{13}C{^{1}H} NMR spectrum of compound **5**. S5

Figure S8. ^{1}H NMR spectrum of compound **6**. S5

Figure S9. ^{13}C{^{1}H} NMR spectrum of compound **6**. S6

Figure S10. ^{77}Se{^{1}H} NMR spectrum of compound **6**. S6

Figure S11. ^{1}H NMR spectrum of PPA (Table 3, entry 4). S7

Figure S12. ^{1}H NMR spectrum of the obtained crystals of *fac*-**4**. S7

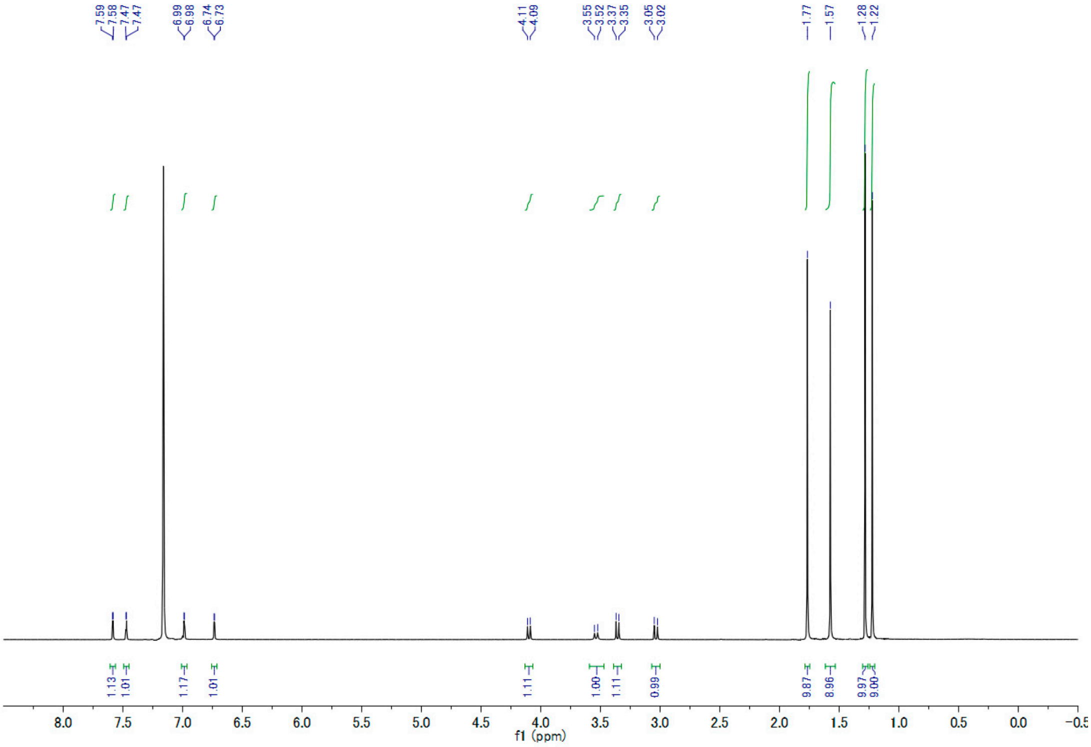

Figure S1. ^{1}H NMR spectrum of compound **3**.

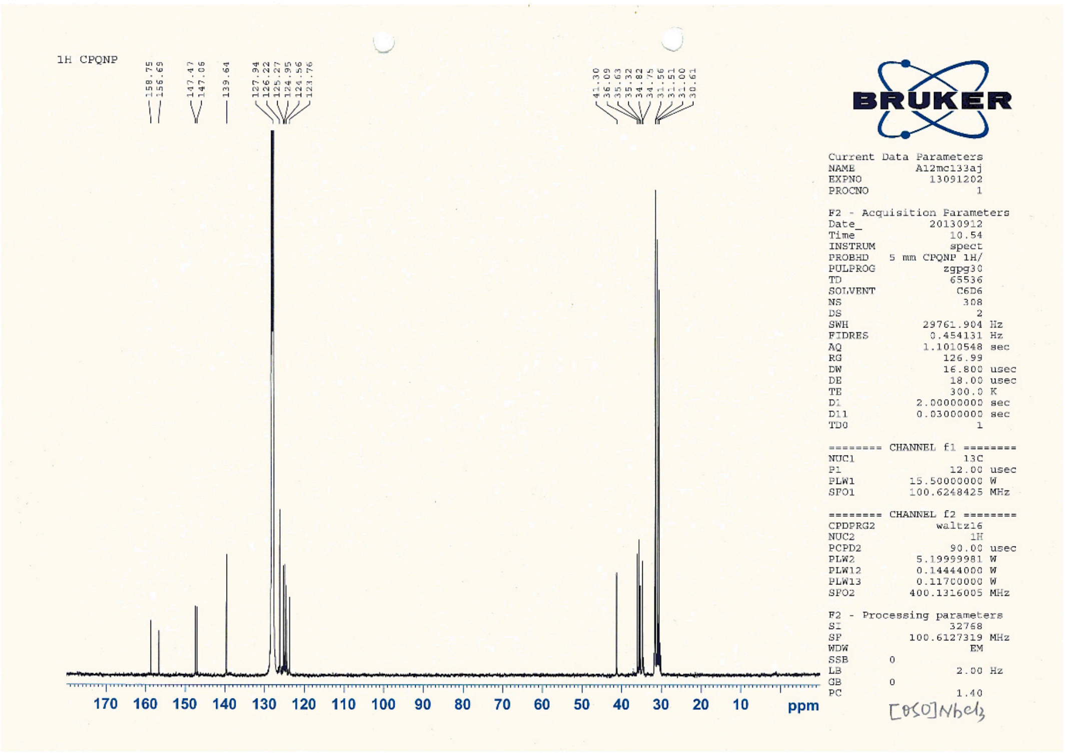

Figure S2. ^{13}C{^{1}H} NMR spectrum of compound **3**.

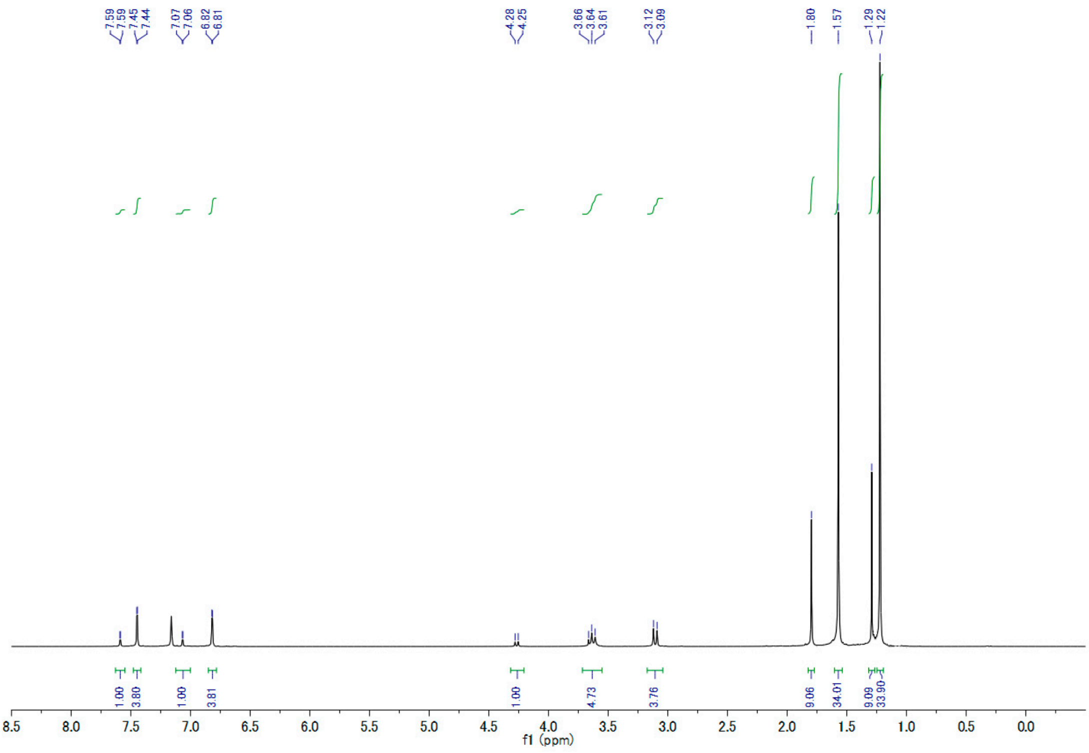

Figure S3. ^{1}H NMR spectrum of compound **4**.

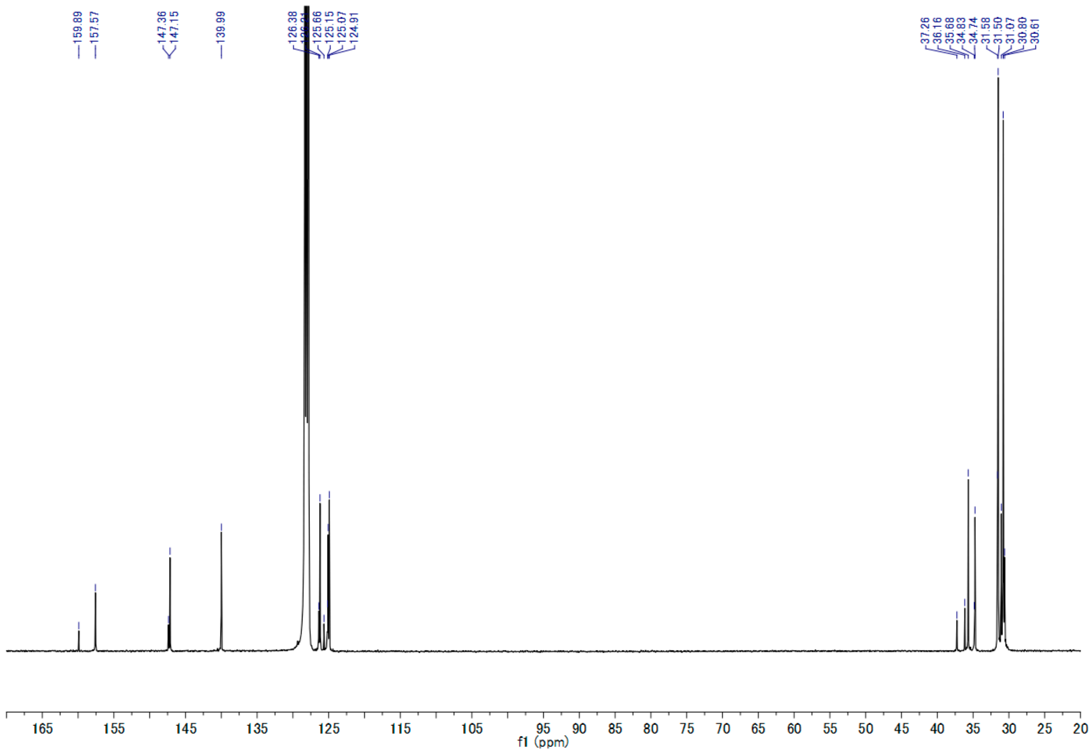

Figure S4. $^{13}C\{^{1}H\}$ NMR spectrum of compound **4**.

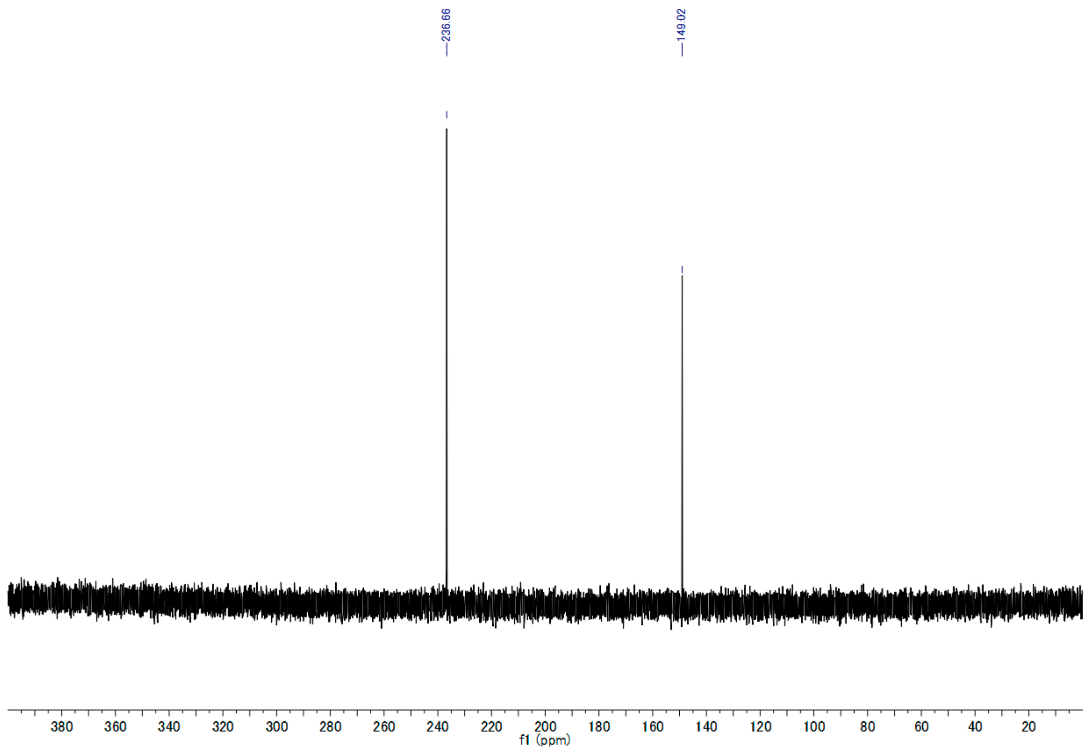

Figure S5. $^{77}Se\{^{1}H\}$ NMR spectrum of compound **4**.

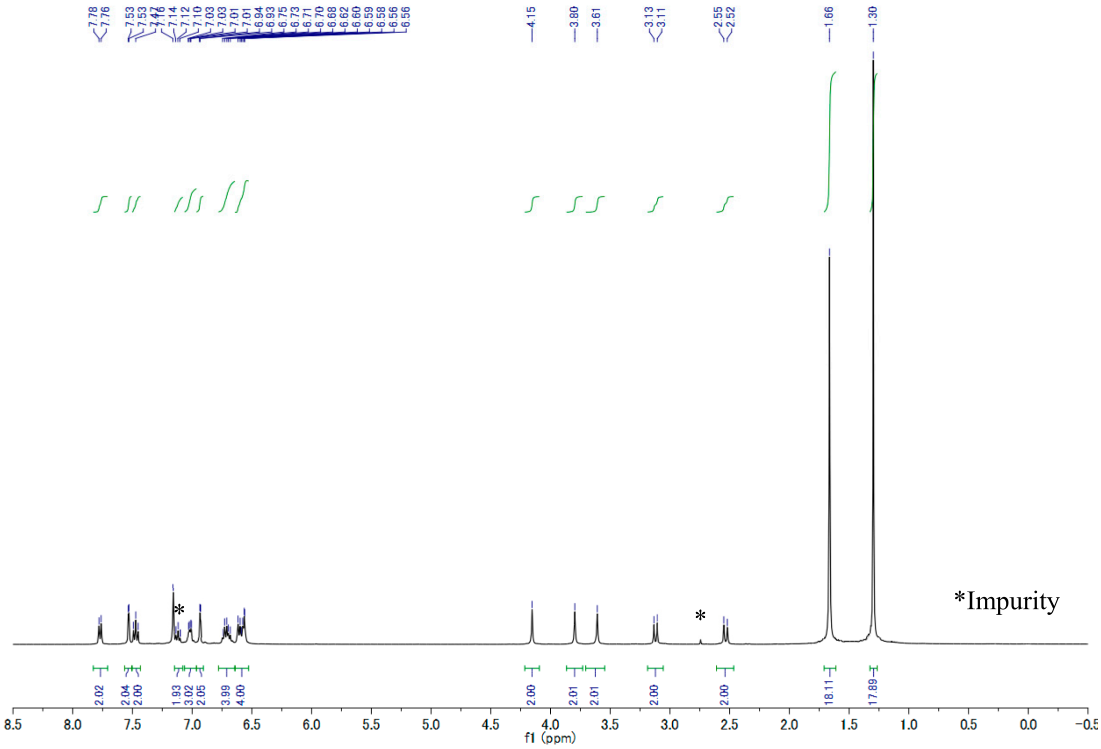

Figure S6. ^{1}H NMR spectrum of compound **5**.

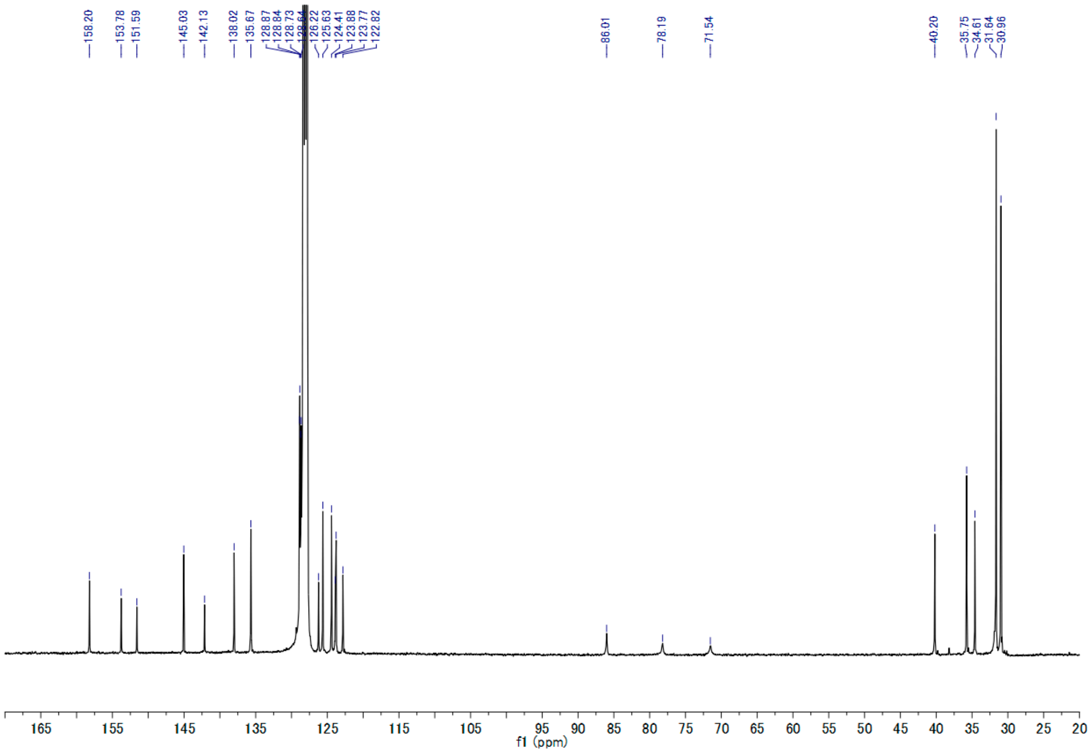

Figure S7. $^{13}C\{^{1}H\}$ NMR spectrum of compound **5**.

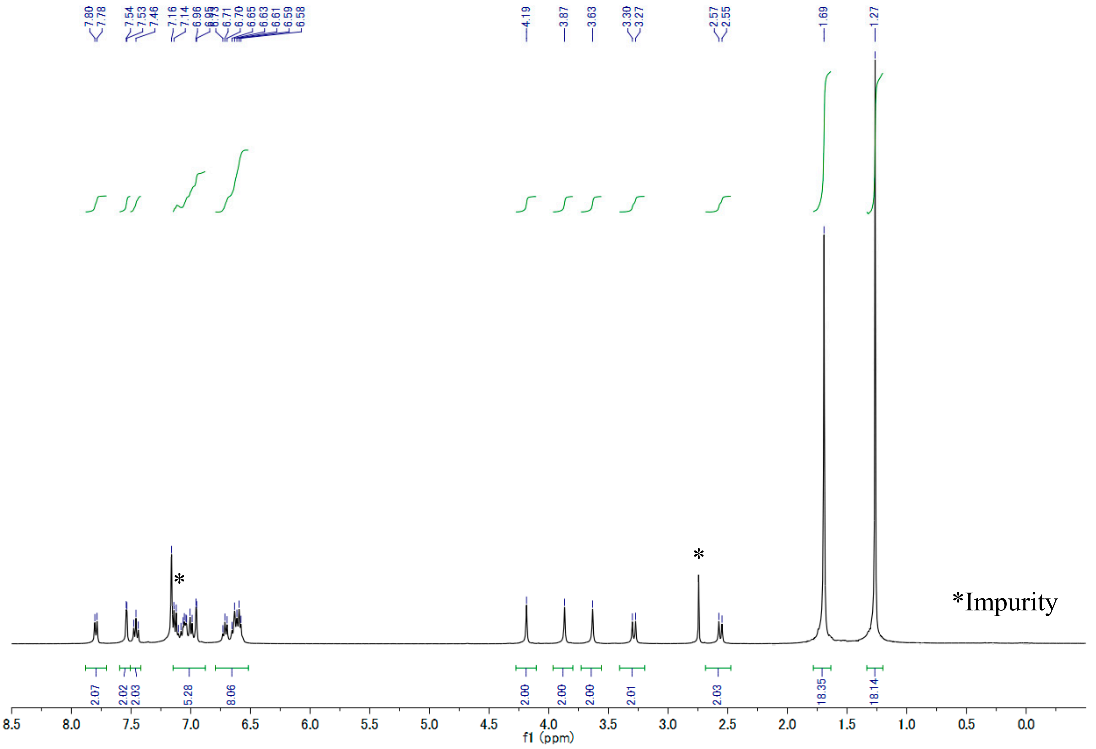

Figure S8. ^{1}H NMR spectrum of compound **6**.

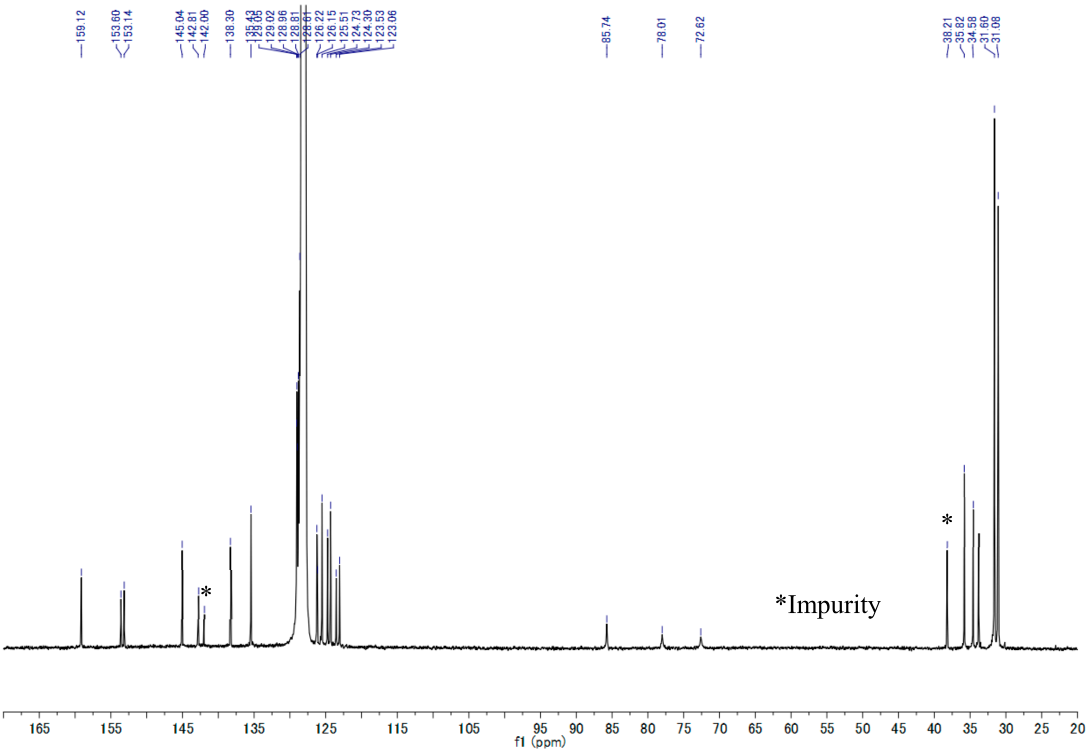

Figure S9. $^{13}C\{^{1}H\}$ NMR spectrum of compound **6**.

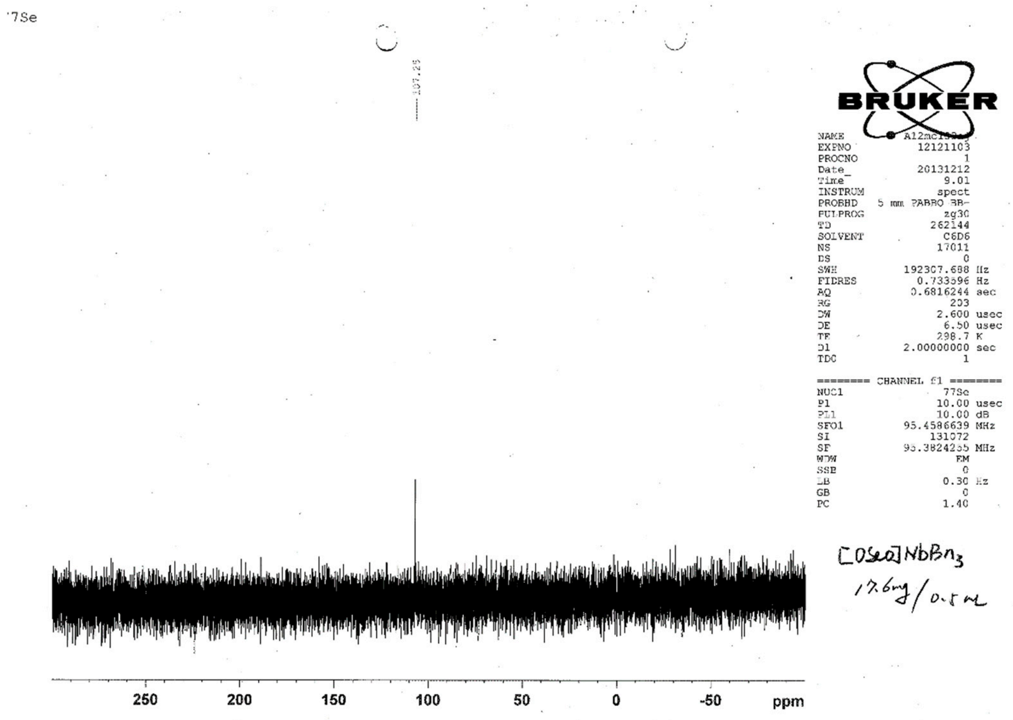

Figure S10. $^{77}Se\{^{1}H\}$ NMR spectrum of compound **6**.

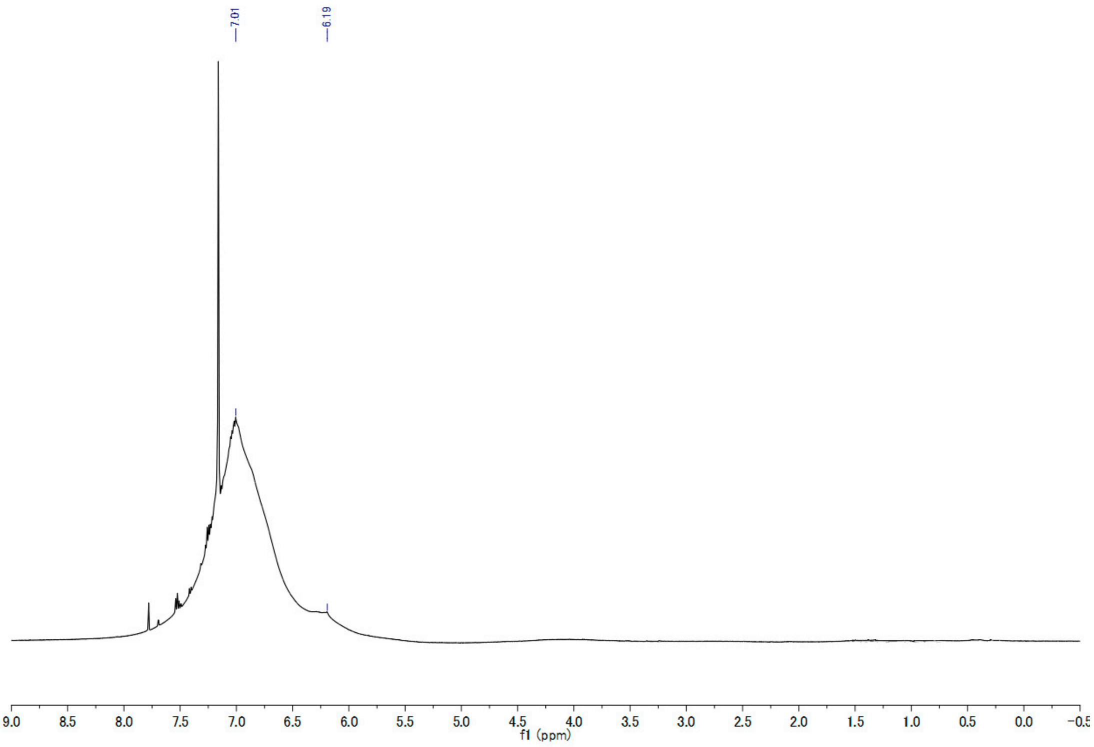

Figure S11. ^{1}H NMR spectrum of PPA (Table 3, entry 4).

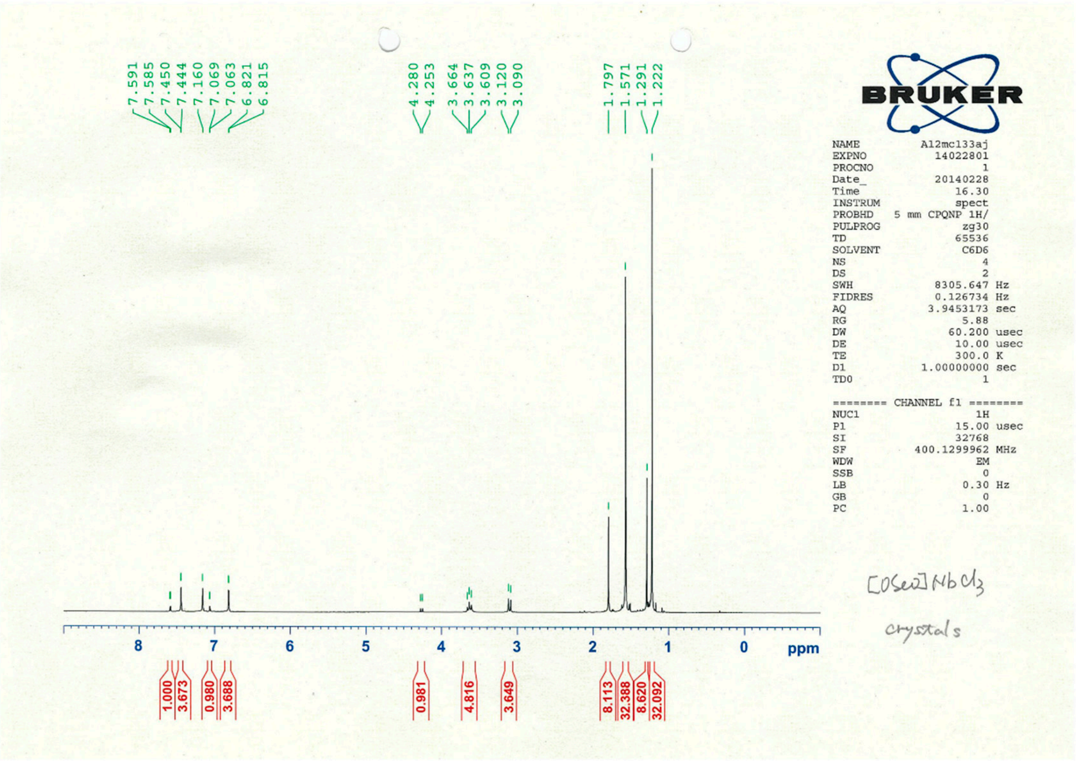

Figure S12. ^{1}H NMR spectrum of the obtained crystals of *fac*-**4**.
